# Comparison of male and female non-refugee immigrants with psychosis: clinical, sociodemographic, and migration-related differences and impact on stress

**DOI:** 10.1007/s00737-024-01431-7

**Published:** 2024-02-19

**Authors:** Amira Trabsa, Francesc Casanovas, Víctor Pérez, Ana Moreno, Benedikt Amann, Anna Mané

**Affiliations:** 1 PhD Programme, Department of Psychiatry and Forensic Medicine, Universitat Autònoma de Barcelona, Barcelona, Spain; 2 Institute of Mental Health, Hospital del Mar, Passeig Marítim de la Barceloneta 25-29, 08019 Barcelona, Spain; 3 Hospital del Mar Medical Research Institute, C/del Aiguader 88, 08003 Barcelona, Spain; 4 Centro de Investigación Biomédica en Red de Salud Mental, Instituto de Salud Carlos III, Barcelona, Spain; 5 Centre Fòrum Research Unit, Hospital del Mar, C/Llull 410, 08019 Barcelona, Spain; 6 Department of Medicine and Life Sciences, Pompeu Fabra University, Barcelona, Spain

**Keywords:** Women, Non-refugee immigrants, Psychosis, Psychological stress, Gender differences, Migration mental health

## Abstract

**Purpose:**

To compare social, clinical, and migration-related factors between male and female immigrants with psychotic disorders and to determine the association between these variables and stress in the last year.

**Methods:**

We administered the Holmes and Rahe Social Readjustment Scale to evaluate psychological stress in 99 non-refugee immigrants (26 women, 73 men) who presented ≥ one psychotic episode (ICD-10 criteria). We compared the two groups in terms of sociodemographic, clinical, cultural, and migration-related variables. A multivariable analysis using a linear regression model (stepwise method) was performed to evaluate potential associations between these variables and stress.

**Results:**

Women were more likely to be married and divorced, had less access to welfare payments, and lower unemployment and homeless rates than men. The most common psychiatric diagnosis was psychosis not otherwise specified with more women being affected (61.5% in women vs. 45.2% in men), but the diagnosis of schizophrenia was more common in men (38.4% vs 15.4%). Both groups exhibited very high levels of stress in the past year (mean total distress score > 300). In women, stress was significantly associated with age at first migration and be a racialized person. By contrast, among men stress was significantly associated with language barrier and comorbidity with a physical disorder.

**Conclusions:**

The results of this study reveal important differences between men and women immigrants. These findings underscore the importance of understanding how gender-specific roles and social expectations intersect with the timing and nature of migration to influence stress levels differently in immigrant women and men with psychotic disorders.

**Supplementary Information:**

The online version contains supplementary material available at 10.1007/s00737-024-01431-7.

## Introduction

International migration is a major global trend. According to data from the United Nations, a total of 280.6 million people migrated worldwide in the year 2020, with women constituting 48.1% of this population, which underscores the pronounced gender dimension within this intricate landscape (IOM- United Nations 2020).

Migration, which is widely recognized as a chronic environmental stressor, requires continuous adjustment to new surroundings (Bustamante et al. 2018). This includes pre-migration trauma, loss of social support, displacement from home, and experiences of social or racial discrimination and so on (Bustamante et al. 2018; Fortuna et al. 2008). Stress is a multifaceted process in which environmental factors trigger physical/psychological responses, requiring individuals to adapt to the evolving environment (Rahe et al. 1970). When adaptation resources are overwhelmed, mental disorders can arise (Gatt et al. 2019; Steel et al. 2016; Rahe et al. 1970). Socioeconomic adversity, such as unemployment or homelessness, has consistently shown correlations with poorer psychotic disorder outcomes, including prolonged undetected psychosis (Hatzimanolis et al. 2020; Peralta et al. 2022; Samele et al. 2001).

Unsurprisingly, a worse mental health has been described in immigrants (WHO 2021). In fact, several meta-analyses have found that migrant populations present a higher incidence of psychotic disorders (RR > 2) (Bourque et al. 2011; Cantor-Graae and Selten 2005; Selten et al. 2019). Interestingly, recent studies have found an association between adverse experiences throughout the migration process and psychosis development (Stilo et al. 2017; Tarricone et al. 2021). Likewise, extreme adversity conditions during migration (such as illegal status or involuntary migration) significantly elevate the risk of psychosis, contribute to a more complex clinical presentation, and reduce recovery likelihood (Golay et al. 2019).

Compared to men, immigrant women with psychotic disorders could face several unique stressors related to their various minority identities (Gea-Sánchez et al. 2017). To understand this, an essential concept comes into play: intersectionality. This term, from sociology, is used to describe the impact and complex interconnection of multiple identities and forms of oppression on experiences of inequality (Choo and Ferree 2010). Immigrant women with psychotic disorders experience a complex interweaving of different social factors such as gender role demands, social stigma related to ethnicity and the psychosis diagnosis, and isolation. These factors could increase the risk of poorer health (Brodish et al. 2011; Gea-Sánchez et al. 2017).

Although it seems clear that women immigrants face a unique combination of stressors, there is an important gap in the literature when it comes to identifying factors linked to stress in immigrant women (Perry et al. 2013; Ryan et al. 2021). The lack of research is particularly notable in immigrant populations with psychotic disorders (Trabsa et al. 2023, Mazza et al. 2021). This issue is especially significant given that vulnerable population groups, who commonly require more healthcare attention, often receive the least support (Fiscella and Shin 2005).

In this context, the aims of this study were (1) to describe and compare the social, clinical, and migration-related variables in immigrant women and men with psychotic disorders and (2) to determine potential associations between those variables and stress in the last year in this population.

## Methods

### Participants

A total of 99 non-refugee immigrants (26 women, 73 men) were recruited between June 2020 and July 2021. Participants were recruited from inpatient and outpatient units at the mental health service at the Hospital Del Mar in Barcelona, Spain. This hospital has a high proportion of immigrants in its catchment area: 41%, without considering undocumented immigrants.

The participants were recruited by convenience sampling. Inclusion criteria were as follows: (1) age 18 to 65 years; (2) confirmed diagnosis of a psychotic disorder according to the International Classification of Diseases 10th revision (ICD-10) (World Health Organization 2004), including any of the following: F.29 nonspecific (not otherwise specified; NOS) psychosis, F.22 delusional disorder, F.25 schizoaffective disorders, or F.20 schizophrenia. Exclusion criteria include: (1) current acute psychotic episode, (2) psychosis secondary to acute intoxication or organic condition, (3) moderate cognitive impairment; (4) existence of significant language barrier (inability to understand English/Spanish/French).

### Data and instruments

Migration process details, along with sociodemographic and clinical data, were obtained using a custom questionnaire and medical records. Validated scales were used to assess clinical and stress-related variables. All interviews were conducted in one of three languages (Spanish, English, or French) in accordance with the participant’s preference. When necessary, the interviews were conducted by a bilingual evaluator trained in cultural competence.

The Holmes and Rahe Social Readjustment Scale (Holmes and Rahe 1967) was used to assess stressful events in the last year, along with Spanish (González de Rivera JL. 1983) and French (Harmon et al. 1970) validated versions. This 43-item scale measures a range of different stressful events, with each event given an impact score. Two measures are used to divide the overall scores: the count of total stressful events and the global impact score. Total impact scores are interpreted as follows: < 150: low stress levels; 150 to 299: moderate stress (with a 50% risk of developing an illness in the near future); and > 300: high stress (illness risk of 80% risk in the near future) (Blasco-Fontecilla et al. 2012; Rahe et al. 1970). For the Spanish version, Gerst et al. (1978) assessed the reliability of the Holmes and Rahe Social Readjustment Scale, reporting high consistency in rank ordering for both healthy adults (ranging from 0.96 to 0.89) and patients (ranging from 0.91 to 0.70). In terms of validity, Holmes and Rahe (1967) identified a positive correlation (+ 0.118) between Life Change scores and illness scores. Furthermore, the scale has been validated and utilized within Spanish populations (Roca et al. 2013). Regarding the French version, Harmon et al. administered the Holmes and Rahe Social Readjustment Scale to French, Belgian, and Swiss samples in a French language translation. Their findings indicated a high concordance among all European samples, with Rho’s of 0.93, 0.94, and 0.96. Additionally, a composite European sample (*n* = 139) compared to a corresponding American sample (*n* = 195) showed a high correlation in relative rank ordering of readjustment required by life events (rho = 0.89)(Harmon et al. 1970). However, all these validations were studied primarily within western context, comparing Europeans and Americans.

The Positive and Negative Syndrome Scale (PANSS), Spanish (Peralta Martín and Cuesta Zorita 1994), English (Kay et al. 1987), and French (Lançon et al. 1999) versions were administered to assess psychotic symptoms. The Spanish version demonstrated good interrater reliability (ICC = 0.72 to 0.80) and moderate internal consistency (*α* = 0.62 to 0.92) (Peralta Martín and Cuesta Zorita 1994). Next, the English version displayed internal consistency ranging from *α* = 0.73 to 0.83 (Kay et al. 1987) and interrater reliability between 0.83 and 0.87 (Kay et al. 1988). Lastly, the French version (Lançon et al. 1999) exhibited Cronbach’s alpha coefficients exceeding 0.65 across the five studied factors: negative, positive, hostility, disorganization, and anxiety/depression. Accordingly, the PANSS can be used to reliably measure schizophrenia symptoms in patients from different cultural and linguistic backgrounds. However, it is not specifically validated for migrant populations, which might influence the accuracy of symptom assessment*.* The Mini-Mental State Examination (MMSE) (Folstein et al. 1975) was administered to detect individuals with a moderate cognitive impairment (MMSE > 15) as this constituted one of the exclusion criteria.

### Ethics

This study was approved by the *Clinical Research Ethics Committee at the Parc de Salut Mar*, Barcelona (ID:2019/8398/I) under the principles laid out in the Declaration of Helsinki (WMA Declaration of Helsinki 2022). Participation was entirely voluntary. All participants were required to provide signed informed consent (available in Spanish/English/French) prior to participation. The study protocol was registered at ClinicalTrials.gov (NCT04867447).

### Data analyses

A cross-sectional descriptive analysis was conducted. Two separate groups (women and men) were constructed for the univariate analyses. Comparative analyses were performed using the *χ* 2 test and Student’s *t* test. Next, Pearson’s correlation coefficients and Student’s *t* test were determined to examine potential associations between stressful events in the past year and sociodemographic, clinical, and migration-related variables. Nominal variables with more than two categories were dichotomized.

A multivariable analysis was performed using a linear regression model (step-wise method). In this model, the total number of events score from the Holmes and Rahe scale was used as the dependent variable. For this analysis, the independent variables, selected based on previous reports (Bustamante et al. 2018; Erving 2022; Sangalang et al. 2018), were as follows: age; education (years); number of migrations; age at first migration; PANSS total positive/negative/general symptoms; racialized; origin; single; living alone; descendants(children); jobless; homeless; non-affective psychosis; comorbid psychiatric history; family psychiatric history; substance use; illegal status; irregular migration transportation; solo migration; and language barrier. *P* values ≤ 0.05 were considered statistically significant. The IBM Statistical Package for Social Sciences (SPSS) (IBM Corp.; Armonk, NY, USA) was used to perform all statistical analyses.

## Results

### Comparison of sociodemographic between women and men

There was a significant between-group (women vs. men) difference in relationship status (*χ*^2^_3_ = 8.2, *p* = 0.04). While most of the sample (70.7%) were single, a significantly higher percentage of women were married (26.9% vs. 19.2%). Similarly, the percentage of divorced women was also higher (15.4% vs. 4.1%). A higher proportion of women had children (46.2% vs. 21.9%) (*χ*^2^_96_ = 9.8, *p* = 0.003). Nearly one in four men (24.7%) were homeless vs. 6.8% of women (*χ*^2^_8_ = 20.21, *p* = 0.02). Significant between-group differences were also found in employment status (*χ*^2^_7_ = 18.1, *p* = 0.02), with a higher proportion of women employed part-time (3.8% vs. 2.7%) or full-time (15.4% vs. 2.7%). By contrast, a higher percentage of the men were receiving a welfare allowance for health-related problems (8.2% vs. 3.8%) (Table 1).

**Table 1:** Comparison of sociodemographic characteristics between immigrant women and immigrant men with psychotic disorders. Data are presented as means (SD) or number (%)

Variable		Group	Obs/Freq	Mean (SD), conf/percentage*	Contrast statistics
Age		Women	26	38.3 (12.7)	*t*_96_ = 9.8 *p* = 0.003
		Men	73	31.6 (8.5)	
		Total	99	33.3 (10.2)	
Education, years		Women	26	10.6 (3.1)	*t*_96_ = 2.4 *p* = 0.2
		Men	73	9.5 (4.2)	
		Total	99	9.8 (3.9)	
Relationship status	Single	Women	14	53.8%	*X*^2^_3_ = 8.2 *p* = 0.04
		Men	56	76.7%	
		Total	70	69.3%	
	Married/in a couple	Women	7	26.9%	
		Men	14	19.2%	
		Total	21	20.8%	
	Widowed	Women	1	3.8%	
		Men	0	0%	
		Total	1	0.99%	
	Divorced/separated	Women	4	15.4%	
		Men	3	4.1%	
		Total	7	6.9%	
Descendants (children)	Yes	Women	12	46.2%	*X*^2^_1_ = 5.6 *p* = 0.02
		Men	16	21.9%	
		Total	28	27.7%	
	No	Women	14	53.8%	
		Men	57	78.1%	
		Total	71	70.3%	
Household	Alone	Women	4	4.0%	*X*^2^_8_ = 20.21 *p* = 0.02
		Men	9	7.1%	
		Total	13	12.9%	
	Family/friends/couple	Women	19	76.7%	
		Men	65	63.5%	
		Total	84	83.2%	
	Mental health residence	Women	1	3.8%	
		Men	1	1.4%	
		Total	2	1.98%	
	Homeless	Women	1	3.8%	
		Men	18	24.7%	
		Total	19	18.8%	
Employment status	Student	Women	2	7.7%	*X*^2^_7_ = 18.1, *p* = 0.02
		Men	2	2.7%	
		Total	4	3.9%	
	Full-time employment	Women	4	15.4%	
		Men	3	2.7%	
		Total	7	6.9%	
	Part-time employment	Women	1	3.8%	
		Men	2	2.7%	
		Total	3	2.9%	
	Sick leave	Women	2	7.7%	
		Men	1	1.4%	
		Total	3	2.9%	
	Unable to work and receiving welfare allowance for health problems	Women	1	3.8%	
		Men	6	8.2%	
		Total	7	6.9%	
	Unemployed	Women	14	53.8%	
		Men	55	75.3%	
		Total	69	68.3%	
	Homemaker	Women	2	7.7%	
		Men	0	0%	
		Total	2	1.98%	

Abbreviations: Obs/Freq, number of cases observed/frequency, *SD* standard deviation

*Age and education data are presented as means. The other variables are given as percentages

### Migration process and cultural variables

A total of 36 countries were represented in the sample, indicating substantial diversity in this group. However, there were significant between-group differences in the country of origin (*χ*^2^_8_ = 17.4, *p* = 0.04), with most of the women from South America (30.8%) or Western Europe (23.1%) and most of the men from North Africa (31.5%) or South America (20.5%). Significant differences were also observed in terms of race (*χ*^2^_3_ = 11.6, *p* = 0.02), with a substantially higher percentage of men classified as black (20.5% vs. 11.5%) and a higher proportion of women classified as Asian (15.4% vs 1.4%). Significant differences were also observed in terms of the method of transportation, with 32.9% of men entering the country illegally versus only 3.8% of women (*χ*^2^_3_ = 8.5, *p* = 0.002). In terms of the legal status, 42.5% of the men were illegal immigrants vs. 3.8% of the women (*χ*^2^_3_ = 13.1, *p* = 0.00) (Table 2).

**Table 2:** Comparison of migration and cultural variables between immigrant women and men with psychotic disorder. Data are presented as means (SD) or number (%)

Variable		Group	Obs/Freq	Mean (SD), conf/percentage*	Contrast statistics
Age at first migration		Women	26	21.9 (10.5)	*t*_96_ = 0.8 *p* = 0.4
		Men	73	19.9 (8.3)	
		Total	99	20.4 (8.8)	
Total number of migrations		Women	26	1.5 (1.5)	*t*_96_ = 1.8 *p* = 0.4
		Men	73	1.8 (1.4)	
		Total	99	1.7 (1.4)	
Total years since first migration		Women	26	16.4 (15.5)	*t*_96_ = 3.17 *p*. = 0.09
		Men	73	11.6 (11.1)	
		Total	99	12.8(12.5)	
Origin	North Africa	Women	1	3.8%	*X*^2^_8_ = 17.4, *p* = 0.04
		Men	23	31.5%	
		Total	24	23.7%	
	Africa	Women	2	7.7%	
		Men	10	13.7%	
		Total	12	11.8%	
	South America	Women	8	30.8%	
		Men	15	20.5%	
		Total	23	22.8%	
	Central America	Women	1	3.8%	
		Men	0	0%	
		Total	1	0.99%	
	North America	Women	0	0%	
		Men	1	1.4%	
		Total	1	0.99%	
	Eastern Asia	Women	2	7.7%	
		Men	6	1.4%	
		Total	8	7.9%	
	Southeast Asia	Women	3	11.5%	
		Men	4	5.5%	
		Total	7	6.9%	
	Middle East	Women	2	7.7%	
		Men	6	8.2%	
		Total	8	7.92%	
	Eastern Europe	Women	1	3.8%	
		Men	6	8.2%	
		Total	7	6.9%	
	Western Europe	Women	6	23.1%	
		Men	7	9.6%	
		Total	13	12.9%	
Race	Caucasian	Women	10	38.5%	*X*^2^_3_ = 11.6, *p* = 0.02
		Men	42	57.5%	
		Total	52	51.5%	
	Black African	Women	3	11.5%	
		Men	15	20.5%	
		Total	18	17.8%	
	Asian	Women	4	15.4%	
		Men	1	1.4%	
		Total	5	4.9%	
	Southeast Asian	Women	2	7.7%	
		Men	5	6.8%	
		Total	7	6.9%	
	American	Women	7	26.9%	
		Men	10	13.7%	
		Total	17	16.8%	
Religion	Christianism	Women	12	46.2%	*X*^2^_3_ = 10.9, *p* = 0.03
		Men	17	23.3%	
		Total	29	28.7%	
	Islam	Women	3	11.5%	
		Men	34	46.6%	
		Total	37	36.6%	
	Buddhism	Women	1	3.8%	
		Men	2	2.7%	
		Total	3	2.9%	
	Atheism	Women	8	30.8%	
		Men	14	19.2%	
		Total	22	21.8%	
	Others	Women	2	7.7%	
		Men	6	8.2%	
		Total	8	7.9%	
Legal status: Undocumented		Women	1	3.8%	*X*^2^_3_ = 13.1, *p* = 0.00
		Men	31	42.5%	
		Total	32	31.7%	
Transportation during migration: Illegal		Women	1	3.8%	*X*^2^_3_ = 8.5, *p* = 0.002
		Men	24	32.9%	
		Total	25	24.7%	
Companions during migration process	Alone/solo	Women	10	38.5%	*X*^2^_1_ = 2.6, *p* = 0.27
		Men	40	54.8%	
		Total	50	49.5%	
	With family or friends	Women	16	61.5%	
		Men	32	43.8%	
		Total	48	47.5%	
Reason for migration	Economical	Women	23	88.5%	*X*^2^_1_ = 10.9, *p* = 0.05
		Men	58	79.5%	
		Total	81	81.8%	
	Political	Women	1	3.8%	
		Men	9	12.3%	
		Total	10	10.1%	
	Studies	Women	2	7.7%	
		Men	6	8.2%	
		Total	8	8.08%	
Language barrier on arrival: yes		Women	15	57.7%	*X*^2^_1_ = 2.9, *p* = 0.07
		Men	55	75.3%	
		Total	70	69.3%	
Current language barrier: yes		Women	6	23.1%	*X*^2^_1_ = 2.3, *p* = 0.12
		Men	29	39.7%	
		Total	35	34.65%	

Abbreviations: Obs/Freq number of cases observed/frequency, *SD* standard deviation

^*^Age and education data are presented as means. The rest of the variables are presented as percentages

### Clinical data and stress comparison between women and men

Significant between-group differences were observed in the main psychiatric diagnosis (*χ*^2^_3_ = 9.8, *p* = 0.02). The most common primary psychiatric diagnosis in both groups was psychosis NOS (61.5% in women vs. 45.2% in men). However, 38.4% of the men with a psychotic disorder were diagnosed with schizophrenia compared to only 15.4% of the women (Table 3).

**Table 3:** Comparison of clinical data and stress levels in the past year between immigrant women and men with psychotic disorder. Data are presented as means (SD) or number (%)

Variable		Groups	Obs/Freq	Mean(SD), conf/percentage*	Contrast statistics
Main diagnosis	Schizophrenia	Women	4	15.4%	*X*^2^_3_ = 9.8 *p* = 0.02
		Men	28	38.4%	
	Schizoaffective disorder	Women	4	15.4%	
		Men	12	16.4%	
	Psychosis NOS	Women	16	61.5%	
		Men	33	45.2%	
	Delusional disorder	Women	2	7.7%	
		Men	0	0.0%	
Comorbid psychiatric diagnosis: yes		Women	2	7.7%	*X*^2^_1_ = 1.2 *p* = 0.271
		Men	2	2.7%	
Physical comorbidity: yes		Women	1	3.8%	*X*^2^_1_ = 0.003 *p* = 0.9
		Men	3	4.1%	
Family psychiatric history: yes		Women	9	34.6%	*X*^2^_1_ = 0.21 *p* = 0.7
		Men	29	39.7%	
Suicide attempts: yes		Women	1	3.8%	*X*^2^_1_ = 3.5 *p* = 0.06
		Men	14	19.2%	
Current psychoactive substance use: yes		Women	5	19.2%	*X*^2^_1_ = 4.8, *p* = 0.03
		Men	31	42.5%	
Past psychoactive substance use: yes		Women	9	34.6%	*X*^2^_1_ = 4.5, *p* = 0.03
		Men	43	58.9%	
Holmes and Rahe	Number of events	Women	26	9.1 (4.2), − 4.5–0.5	*t*_96_ = 0.6, *p* = 0.02
		Men	73	11.6 (4.4), − 4.5–0.5	
	Total distress score	Women	26	301.2 (4.2), − 137.6–11.6	*t*_96_ = 0.02, *p* = 0.02
		Men	73	375.6 (139.1), − 137.6–11.6	
PANSS	Positive symptoms	Women	26	14.2 (7.4), − 1.01–3.84	*t*_96_ = 12.9, *p* = 0.13
		Man	73	12.7 (4.4), − 1.73–4.5	
	Negative symptoms	Women	26	13.2 (6.7), − 5.2–2.4	*t*_96_ = 1.27, *p* = 0.24
		Man	73	14.6 (8.9), 4.7–1.9	
	General symptoms	Women	26	28.9(11.1), − 3.8–5.1	*t*_96_ = 1.06, *p* = 0.39
		Man	73	28.3 (9.4), − 4.3–5.6	

Abbreviations: Conf. confidence, Psychosis, *NOS *Psychosis Not Other Specified, *PANSS* Positive and Negative Syndrome Scale. Holmes and Rahe Holmes and Rahe Social Readjustment Scale

*Scale data are presented as means. The other variables are presented as percentages

Significant differences were also observed on the Holmes-Rahe Life Stress Inventory. Although stress levels were high in both groups, they were significantly higher in men, as evidenced by the mean total distress score of 375.6 in men vs. 301.2 in women (*t*_96_ = 0.02, *p* = 0.02) and an average of 11.6 stressful events in the last year in men vs. 9.1 events in women (*t*_96_ = 0.6, *p* = 0.02) (Table 3).

### Associations between stress in the past year and sociodemographic, clinical, and migration variables

On the bivariate analysis, women show association with age at first migration and stress in the past year. In men this association was seen with comorbid diagnosis, illegal status, illegal transportation, migration solo, and language barrier (Supplementary material 1).

On the multivariate analysis, the model that best predicted stress in the past year in women was a two-variable model (age at first migration and be a racialized person), with an adjusted *R*^2^ of 0.186. By contrast, in men the best predictor of stress was a four-variable model (PANSS total score, comorbidity with a physical disorder, irregular transportation, and language barrier), with adjusted *R*^2^ of 0.182. For women, a lower age at first migration and be a racialized person were significantly associated with higher stress levels in the past year (see Table 4). In men group, comorbid a physical diagnosis and language barrier were significantly associated with stress. Irregular transportation and general PANSS symptoms presented an important association tendency (Table 4).

**Table 4:** Linear regression model for stress in the past year measured by total number of events reported on the Holmes and Rahe scale (dependent variable)

Women				
	B	*t*	*p*	Confidence interval
Age at first migration	− 0.14	− 1.9	0.05	− 0.29–0.01
Racialized	2.9	1.9	0.06	− 0.17–6.15
Men				
	B	*t*	*p*	Confidence interval
PANNS general symptoms	− 0.01	− 1.88	0.06	− 0.19–0.01
Comorbid physical diagnosis	2.48	1.98	0.05	0.01–4.98
Illegal transportation	− 2.08	− 1.88	0.06	− 4.29–0.12
Language barrier	2.09	1.99	0.05	0.00–4.19

*PANSS* positive and negative syndrome Scale, Holmes and Rahe Holmes and Rahe Social Readjustment Scale

## Discussion

The results of this study reveal significant differences between immigrant women and men with psychotic disorders in terms of sociodemographic, clinical, and migration process-related variables. Significant between-group differences were also observed in terms of the association between these variables and stress levels over the past year.

### Comparison of sociodemographic between women and men

First, while the predominant origin among men aligns with the main source of migration to Spain (11.49% of migrants in Spain are from Morocco) (IOM- United Nations 2020), most women in our study are from South America. Although current evidence suggests a slightly higher prevalence of psychosis in men compared to women (Jongsma et al. 2019), the incidence of psychosis appears to remain consistent worldwide (0.7–1%) (Jauhar et al. 2022; McGrath et al. 2008). Given these data, it becomes evident that there is an under-representation of North African women in our sample, which prompts next question: why are these women not accessing our services? It has been well described, that immigrants, due to different barriers, have less access to specialized, primary and mental healthcare when compared to locals (Fiscella and Shin 2005; Pérez-Urdiales 2021). These differences are even more pronounced among immigrant women (Gea-Sánchez et al. 2017). In this regard—and in line with our results—a previous study carried out in Spain found that Latin women accessed the healthcare system more frequently than African women, mainly due to cultural and linguistic similarities with the host culture (Pérez-Urdiales 2021).

In our sample, 46.2% of the women had children versus only 21.9% of men. Interestingly, more than half of these women were single (53.8%). These findings shed light into a critical factor that have been described to influence psychosis risk among immigrants (Dykxhoorn et al. 2019). Previous research describe that women immigrating alone appear to have a higher risk of psychosis than those immigrating with their families (Dykxhoorn et al. 2019). Conversely, among men, those immigrating to join a family and those immigrating with their dependent children had an increased risk of psychosis (Dykxhoorn et al. 2019). Thus, sex-specific differences in how family networks are perceived during the migration process and the association with psychosis risk must be considered.

Another notable gender-related difference was that the divorce/separation rate was nearly three times higher in women than in men. This finding is consistent with previous research showing that women facing serious medical illness are more likely to be abandoned by their partners (Glantz et al. 2009). Furthermore, when partner separation occurs it leads to a negative impact on life’s quality (Glantz et al. 2009). While previous research did not focus on psychosis, it is well known that women who live with psychosis deal with important social stigma (Chernomas et al. 2017). Another concerning finding was the relatively low percentage of women receiving a welfare allowance compared to men (3.8% vs. 8.2%). The increased welfare allowances among immigrant men cannot be solely explained by worse symptomatology according to PANSS scores. The reason for this difference is not clear, but this finding warrants more study to prevent gender discrimination.

Even though intersectional factors create a more challenging situation for female immigrants, women had a substantially higher employment rate than men (15.4% vs. 2.7%). This finding—considered together with the lack of between-group differences in terms of education and PANSS scores—is consistent with the findings from other studies describing the “feminization of survival”, in which female immigrants take on a larger role than men in providing for their families (González-Juárez et al. 2014). However, it is important to note that a higher proportion of men present an illegal status, which may also contribute to these lower job rates.

### Clinical data and stress comparison between women and men

Regarding clinical variables, women, compared to men, were diagnosed more frequently with psychosis NOS and less with Schizophrenia. This finding may be explained by insights from prior research studies. First, cultural barriers, together with insufficient cultural competence in healthcare services, could lead to a less precise diagnosis (Alda Díez et al. 2010; Bhui et al. 2007). In this regard, some studies suggest that the underrepresentation of minority perspectives in academia may lead to the development of ethnocentric mental health assessment protocols (Aggarwal et al. 2016; Dein 2007). Moreover, the clinical characteristics of psychosis in women (more mood symptoms, better functioning, etc.) could lead—independently of migrant status—to misdiagnosis and treatment delays in this population (Ferrara and Srihari 2020; Mazza et al. 2021). Finally, immigrants present worse adherence to mental health services, which might explain lower diagnostic rates for chronic illnesses such as schizophrenia (Betancourt et al. 2003; Betancourt et al. 2017; Pérez-Urdiales 2021).

When comparing Holmes and Rahe scores, although men presented more distress secondary to stressful events in the past year (impact scores of 375.6 among men vs 302.2 women) both groups presented exceptionally high impact scores. Previous literature have described that impact scores above 300 could result in an upcoming illness risk (physical or mental) of 80% (Blasco-Fontecilla et al. 2012; Rahe et al. 1970). Plus, stress, as measured by Holmes and Rahe scores, significantly influences psychotic symptoms and risk (Butjosa et al. 2016; Myin-Germeys et al. 2005).

### Associations between stress in the past year and sociodemographic, clinical, and migration variables

We performed a multivariable analysis to determine whether any sociodemographic, migration, or clinical variables associated with stressful events or distress in the past year. In women, stress levels were significantly associated with age at first migration (that is, younger migrants report more stress) and be a racialized person (racialized migrants report more stress). By contrast, in men, stress was associated with factors related to the migration process (language barrier) and clinical burden (physical disorder). A potential explanation for these differences could be that stress levels are influenced by traditional gender roles and expectations (Alexander et al. 2021), in which the responsibility of taking care of the family falls mainly on women (Luo and Sato 2021; Wu et al. 2021). As a result, when women migrate to a new country, they may face challenges adapting to a new environment while simultaneously upholding these traditional family roles (Alexander et al. 2021; Luo and Sato 2021). This dual pressure can lead to increased stress, especially when migration occurs at a critical time in their life, such as early adulthood or young motherhood when they have young children (Wu et al. 2021). This is particularly relevant given the findings recently reported by Wu et al., who found post-migration stress has time-varying effects on mental health (Wu et al. 2021). Moreover, several studies have shown that racism and sexism are interconnected, and individuals with multiple disadvantages face a higher exposure to discrimination, leading to more stress and worse mental health (Denise 2012; Perry et al. 2013; Thomas et al. 2008). This could explain our results, in which being a racialized person was associated with stress only in women.

In contrast, men, face challenges related to migration and adaptation within the framework of their provider and protector roles (Abdullah and Brown 2011; Assari and Lankarani 2017; Gelfer 2014; Wu et al. 2021), resulting in stress that is less influenced by their age at first migration but more in factors that endure migration process itself. For instance, physical illness comorbidity, which could exacerbate disability, burden, and diminish quality of life in individuals with psychosis (Šimunović Filipčić and Filipčić, 2018).

There is, however, a need to move beyond stereotypical and overly simplistic gender models to better understand a highly complex gendered reality (Alexander et al. 2021; Lokot 2018). The explanations underlaying these differences need to be corroborated and studied further.

### Limitations

This study has several limitations. First, recruitment was based on convenience sampling, which could limit the generalizability of the results. Second, there was a notable difference in the number of male and female participants in the study (26 women vs. 73 men), which could limit the statistical power. Third, we employed instruments that are typically standardized for western academia; this may introduce limitations in the cultural applicability. While these tools might demonstrate high validity and reliability within certain cultural contexts, their transferability to diverse cultural settings, such as those represented by immigrants from various regions, might require further validation and adaptation. However, we mitigated this limitation by ensuring that professionals trained in cultural competence performed the evaluations. Fourth, despite the cultural diversity of this sample, the results may not be universally applicable. Our study involved migrants from 36 countries, so we acknowledge the potential influence of migration experiences across regions, contributing to considerable variability among the diverse migrant participants in this study. The adjusted *R*^2^ of 0.186 denotes a low effect size, likely influenced by this diversity, which may have contributed to the modest explanatory power of the model. Fifth, we did not assess resilience, which could be a confounding factor for the impact of stress on the individual. Finally, the cross-sectional design does not allow us to establish causality. Given these limitations, it is important to approach the interpretation of our findings with caution. Further research should focus on cross-cultural validation of assessment tools, investigations across diverse regions, and longitudinal studies to elucidate causal relationships and long-term implications.

## Conclusions

This study reveals significant sociodemographic, clinical, and migration-related differences between male and female immigrants with psychotic disorders. Our findings suggest that the impact of migration-related variables on past year stress differs between male and female immigrants. These findings highlight the high stress burden present in immigrants with psychotic disorders. Immigrant women with psychotic disorders face complex challenges due to the intersectionality of their multiple minority status.

Our findings, considered together with previous reports, indicate that gender-specific roles and social expectations intersect with the timing and nature of migration to influence stress levels differently in male and female immigrants. It is important to develop new policies and interventions that include a gender perspective to mitigate psychological distress in immigrant women with psychotic disorders.

## Supplementary Information

Below is the link to the electronic supplementary material.

Supplementary file1 (DOCX 30 KB)

## Data Availability

The data that support the findings of this study are available on request from the corresponding author, [B.A.], upon reasonable request. The data are not publicly available due to containing information that could compromise the privacy of research participants.
