# Supplementary material for: Comparison of male and female non-refugee immigrants with psychosis: clinical, sociodemographic, and migration-related differences and impact on stress
Source: Arch Womens Ment Health. 2024 Feb 19;27(5):679–92. doi: 10.1007/s00737-024-01431-7 (PMC11405427; doi:10.1007/s00737-024-01431-7)
Supplement: Supplementary file 1 — Supplementary file1 (DOCX 30 KB) [file 737_2024_1431_MOESM1_ESM.docx]

**Supplementary materiaLS**

**Supplementary materials 1**:  Association between stress in the past year measured by scores on the Holmes-Rahe scale* and sociodemographic, clinical, and migration variables in immigrant women and men with psychotic disorder (bivariable analysis).

| **Independent variable** | **Dependent variable**  **(Holmes & Rahe scores)** | **Contrast statistics**  **(correlation r/Student’s t-test)** | | | |
| --- | --- | --- | --- | --- | --- |
|  |  | **Women** | | **Man** | |
|  |  | r | t | r | t |
| **Age** | Total number of events | -0.14 |  | -0.12 |  |
|  | Total distress score | -0.35 |  | -0.61 |  |
| **Education, years** | Total number of events | 0.05 |  | -0.08 |  |
|  | Total distress score | 0.05 |  | -0.14 |  |
| **Total number of migrations** | Total number of events | 0.03 |  | 0.17 |  |
|  | Total distress score | 0.05 |  | 0.22 |  |
| **Age at first migration** | Total number of events | -0.36 |  | -0.12 |  |
|  | Total distress score | -0.40* |  | -0.13 |  |
| **PANSS positive symptoms** | Total number of events | -0.13 |  | -0.12 |  |
|  | Total distress score | -0.14 |  | -0.09 |  |
| **PANSS negative symptoms** | Total number of events | -0.13 |  | 0.05 |  |
|  | Total distress score | -0.12 |  | 0.04 |  |
| **PANSS general symptoms** | Total number of events | -0.25 |  | -0.14 |  |
|  | Total distress score | -0.28 |  | -0.15 |  |
| **Racialized** | Total number of events |  | 2.80 |  | 2.65 |
|  | Total distress score |  | 1.05 |  | 5.92 |
| **Single** | Total number of events |  | 1.13 |  | 4.78 |
|  | Total distress score |  | 1.36 |  | 1.00 |
| **Descendants: yes** | Total number of events |  | 1.57 |  | 2.82 |
|  | Total distress score |  | 2.23 |  | 4.10 |
| **Lives alone** | Total number of events |  | 0.03 |  | 4.48 |
|  | Total distress score |  | 0.06 |  | 3.76 |
| **Jobless** | Total number of events |  | 0.57 |  | 0.09 |
|  | Total distress score |  | 0.40 |  | 0.42 |
| **Non-affective psychosis** | Total number of events |  | 2.38 |  | 0.26 |
|  | Total distress score |  | 2.75 |  | 0.77 |
| **Comorbid psychiatric disorder** | Total number of events |  | 5.43 |  | 2.89 |
|  | Total distress score |  | 1.57 |  | 1.57 |
| **Comorbid organic disorder** | Total number of events |  | 2.03 |  | 1.19* |
|  | Total distress score |  | 6.98 |  | 2.91* |
| **Family history of psychiatric disorder** | Total number of events |  | 0.083 |  | 0.94 |
|  | Total distress score |  | 0.012 |  | 1.01 |
| **Previous suicide attempts** | Total number of events |  | - |  | 0.057 |
|  | Total distress score |  | - |  | 0.05 |
| **Current psychoactive substance use** | Total number of events |  | 1.39 |  | 0.90 |
|  | Total distress score |  | 0.92 |  | 0.32 |
| **Illegal status** | Total number of events |  | - |  | 0.46 |
|  | Total distress score |  | - |  | 0.41* |
| **Illegal transportation** | Total number of events |  | - |  | 2.50* |
|  | Total distress score |  | - |  | 1.46* |
| **Migration alone** | Total number of events |  | 0.11 |  | 2.40* |
|  | Total distress score |  | 0.29 |  | 2.98* |
| **Language barrier** | Total number of events |  | 0.44 |  | 1.93* |
|  | Total distress score |  | 0.11 |  | 2.99* |

*PANSS: Positive and Negative Syndrome Scale.* *Holmes& Rahe: Holmes & Rahe Social Readjustment Scale***P<0.05*

***“Supplementary materials 2: Migration Context in Spain. (C.Finotelli, R.Sebastian, 2023)***

In recent decades, Spain has witnessed substantial immigration, with foreign-born individuals comprising an important portion of its population compared to major destination countries like the UK and the US.

With Spain now hosting a high percentage of foreign-born residents, the demographic landscape has changed significantly from its traditional emigration pattern. In the mid-20th century, there was a noticeable shift in immigrant population, which surged during the early 21st century economic boom, although the subsequent financial crisis did not drastically reduce it.

With time, Spain has developed more inclusive immigration policies, offering extensive civic rights to immigrants, even allowing a path to citizenship. Despite concerns expressed by some radicals-right groups, these policies, aligned with labour market demands and immigrant aspirations, have suffered little political resistance. Spain's substantial improvements in immigration management are obscured by media coverage of irregular migration, creating the perception that the country's immigration policy is characterized by many irregular migrants.

In order to prevent irregular status from becoming an ongoing cycle, the Spanish government takes measures to stabilize the administrative system. To integrate immigrants into the regular labour market and society, policies like the 'Padrón' system (municipal census of residents) are implemented. However, immigrants need to show 3 years of “padrón” to access regularity. The evolution of Spain's immigration laws has moved towards a predictable path for regularization, distinguishing it from models in other countries, and recognizes the contributions of immigrants to the economy while integrating them gradually into society and offering pathways to citizenship.

Spain's demographic change has been remarkable, going from a country of emigration to one of the largest immigrant-receiving nations in Europe. The influx of immigrants has significantly influenced Spain's population growth, accounting for nearly 16% of its total population, which grew from approximately 40 million in 1998 to about 47.5 million in 2022.

Regarding the composition of the immigrant populations, in recent years, while the proportion of immigrants from Africa remained relatively stable, the presence of Latin Americans and Asians has nearly doubled. The share of immigrants from other European countries has diminished compared to 25 years ago. In 2022, the largest groups of immigrants were from Morocco, Colombia, Romania, Venezuela, Ecuador, and Argentina. Notably, the Chinese immigrant population in Spain surpassed that from many EU Member States. Regarding migration patterns, the immigrants arrived primarily for family reunification, employment, retirement (especially from Northern Europe), and, in recent years, asylum-seeking.

This demographic landscape in Spain, reflects how immigrants from diverse origins shape the country's cultural diversity and contribute to Spanish economy and society.”

*C.Finotelli. R.Sebastian. A Pragmatic Bet: The Evolution of Spain’s Immigration System. Migration Policy Institute. April 2023. https://www.migrationpolicy.org/article/spain-immigration-system-evolution.*
